# Mean Hitting Time for Random Walks on a Class of Sparse Networks

**DOI:** 10.3390/e24010034

**Published:** 2021-12-24

**Authors:** Jing Su, Xiaomin Wang, Bing Yao

**Affiliations:** 1 School of Electronics Engineering and Computer Science, Peking University, NO. 5 Yiheyuan Road, Haidian District, Beijing 100871, China; jingsu@pku.edu.cn; 2 Key Laboratory of High Confidence Software Technologies, Peking University, Beijing 100871, China; 3 College of Mathematics and Statistics, Northwest Normal University, Lanzhou 730070, China; yybb918@163.com

**Keywords:** complex network, graphic operation, random walk, mean hitting time, Kirchhoff index

## Abstract

For random walks on a complex network, the configuration of a network that provides optimal or suboptimal navigation efficiency is meaningful research. It has been proven that a complete graph has the exact minimal mean hitting time, which grows linearly with the network order. In this paper, we present a class of sparse networks G(t) in view of a graphic operation, which have a similar dynamic process with the complete graph; however, their topological properties are different. We capture that G(t) has a remarkable scale-free nature that exists in most real networks and give the recursive relations of several related matrices for the studied network. According to the connections between random walks and electrical networks, three types of graph invariants are calculated, including regular Kirchhoff index, M-Kirchhoff index and A-Kirchhoff index. We derive the closed-form solutions for the mean hitting time of G(t), and our results show that the dominant scaling of which exhibits the same behavior as that of a complete graph. The result could be considered when designing networks with high navigation efficiency.

## 1. Introduction

A complex network is recognized as a powerful tool for revealing the mysteries of complex systems [1]. It is widely used in metabolic networks [2], software engineering [3], ecosystems [4] and so on. In addition to some topological parameters, such as power-law degree distribution, average path length and clustering coefficient of complex network, the random walks also received widespread attention because the research of random walk theory can disclose dynamic processes on complex networks. As a key quantity of random walks, the hitting time is related to the mixing rate of an irreducible Markov chain, and it is also considered when calculating the expected time of mixing the Markov chain [5]. The hitting time can be used to measure the navigation efficiency of the network [6,7], and it has a core position in different disciplines, including mathematics, computer, biology, physics, control science and engineering [8,9,10,11,12].

Most of the previous research on random walks about complex networks focuses on two aspects: one is that the nodes of the studied network have identical walking rules [13,14], and the other is to study random walks on heterogeneous networks that set a trap on the node with the largest degree and have scale-free characteristics [15,16,17]. Since many real networks have a scale-free nature, every node in the network can be a trap. Thus, we construct a deterministic network that satisfies the above restrictions to approximate the real network, and it is more beneficial for us to evaluate the dynamic process of the network.

It has been proven that the complete graph has the minimum mean hitting time among all undirected networks, which shows that its propagation is quite efficient [18]. For the purpose of constructing a highly efficient network and controlling its trapping process, it is necessary to explore and design some networks with a small mean hitting time. Since most real networks are sparse networks, the average degree of these networks is much less than that of a complete graph. In this paper, we design and analyze a class of sparse networks with scale-free properties; their topological properties are different from the complete graph. We prove that the dominant scaling of the mean hitting time exhibits the same behavior as that of a complete graph, and they can also have high navigation efficiency.

The main contents in the other sections of this paper are as follows. In Section 2, we propose a graphic operation and design a class of sparse networks, show their differences in several topological parameters, including average degree, degree distribution, clustering coefficient and diameter. In Section 3, we present some lemmas about electrical networks and random walks. In Section 4, we analytically obtain the closed-form solution of the mean hitting time according to the connections between the Kirchhoff index and the mean hitting time. In Section 5, we conclude our work with a concise narrative.

## 2. Topological Characteristics of the Network

Before proceeding, we propose a graphic operation called the rhombus operation and construct a network G(t) by iterating the rhombus operation. Then, we compare the topological features of G(t) and the complete graph $K_{N_{t}}$ with the same network order.

**Figure 1:**
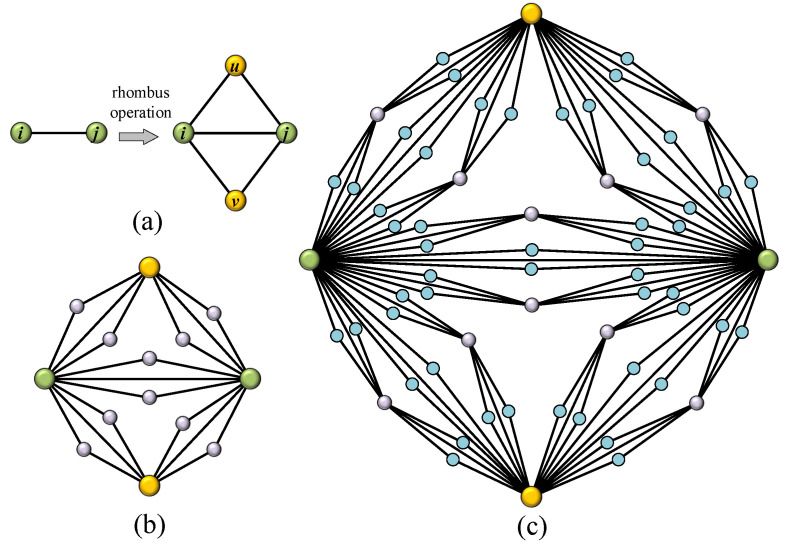
(**a**) The illustration of rhombus operation; (**b**) the network G(t) at time step t=2; (**c**) the network G(t) at time step t=3.

Rhombus operation: For a given edge ij with two endnodes *i* and *j*, add two new nodes to both sides of this edge, denoted by *u* and *v*, and then connect edge ui,uj,vi,vj, respectively. Figure 1a shows the operation process of a rhombus operation.

With the preparation of a graphic operation, we show the construction rule of networks G(t) as follows. Initial state, t=0, G(0) is only an edge. For t≥1, G(t) can be born from G(t−1) by performing a rhombus operation on every edge in G(t−1). Figure 1b,c illustrate the topological structure of G(2) and G(3).

The iterative construction allows us to precisely analyze relevant topological properties of the network. Let V(t) and E(t) be the node set and edge set of G(t); in more detail, the new node set and new edge set at time step *t* are denoted as $\bar{V}\left(t\right)$ and $\bar{E}\left(t\right)$, which means $V\left(t\right)=V\left(t-1\right)\cup \bar{V}\left(t\right)$, and the node belonging to V(t−1) is called the old node. Then, the number of nodes and edges of the network are denoted as $N_{t}$ and $E_{t}$, respectively. The recursive relation $E_{t}=5E_{t-1}$ is obviously established according to the rhombus operation, and we can obtain $E_{t}=5^{t}$ due to $E_{0}=1$. Additionally, we have $N_{t}=N_{t-1}+2E_{t-1}$, so it is easy to verify that $N_{t}=N_{0}+2\sum_{i=0}^{t-1}\;E_{i}=\left(5^{t}+3\right)/2$. Let $k_{i}^{\left(t\right)}$ be the degree of node *i* in G(t) that was generated at iteration $t_{i}$, which satisfies that the degree of node *i* at time step *t* is 3 times the degree of the previous time step t−1; that is, $k_{i}^{\left(t\right)}=3k_{i}^{\left(t-1\right)}$.

### 2.1. Average Degree

**Theorem** **1.** *For the network G(t) with $N_{t}$ nodes and $E_{t}$ edges, the solution of the average degree of network G(t) is*

(1) $$\langle k\rangle =\frac{2E_{t}}{N_{t}}=\frac{4\times 5^{t}}{5^{t}+3}\approx 4.$$

When t→∞, the condition $E_{t}\ll \frac{N_{t}\left(N_{t}-1\right)}{2}$ is clearly established, so our network model is a sparse network according to literature [19]. However, the complete graph is not sparse. For a complete graph $K_{N_{t}}$ with the same number of nodes, the average degree of $K_{N_{t}}$ is $N_{t}-1$ due to the degree of each node is $N_{t}-1$.

### 2.2. Cumulative Degree Distribution

In real life, there are few fully connected networks like a complete graph. Most real networks exhibit scale-free nature, and their nodes with a large degree are fewer, but nodes with a small degree are the majority of the network. A network is said to be scale-free when its cumulative degree distribution obeys $P_{cum}\left(k\right)∼k^{1-\gamma }$, where 2<γ<3, and the cumulative degree distribution $P_{cum}\left(k\right)=\sum_{k^{\prime }=k}^{\infty }P\left(k^{\prime }\right)$ represents the probability that the degree of a node is equal to or greater than *k*, where P(k) is a probability of a randomly selected node with *k* neighbors in the network G(t).

**Theorem** **2.** *The cumulative degree distribution of the sparse network G(t) obeys the following power law distribution,*

(2) $$P_{cum}\left(k\right)=k^{-\frac{\ln5}{\ln3}},\;\;\gamma =1+\ln5/\ln3.$$

**Proof.**  The degree of node *i* will increase by a factor 3, that is, $k_{i}^{\left(t+1\right)}=3k_{i}^{\left(t\right)}$, which shows that the degree spectrum of G(t) is discrete. In Table 1, we enumerate the degree *k* and the number n(k) of nodes with degree *k*, then the cumulative degree distribution of G(t) is calculated by

(3) $$P_{cum}\left(k\right)=\sum_{k^{\prime }=k}^{\infty }\frac{n(k^{\prime })}{N_{t}}=\frac{5^{t_{i}}+3}{5^{t}+3}\approx k^{-\frac{\ln5}{\ln3}},$$

where $t_{i}=t-\frac{\ln k-\ln2}{\ln3}$ has been substituted into the above formula, and for large *t*, the cumulative degree distribution follows a power law $k^{1-\gamma }$ with exponent $\gamma =1+\frac{\ln5}{\ln3}$. Therefore, we have proven that the network G(t) is a scale-free network. On the other hand, the degree of all nodes in the complete graph $K_{N_{t}}$ is the same, so $K_{N_{t}}$ is not a scale-free network. It can be seen that our network is more suitable for simulating scale-free real networks. □

### 2.3. Clustering Coefficient

The clustering coefficient is used to describe the tightness of clumps between nodes in the graph. Specifically, it is a measure of the dense or sparse connection between neighbors of each node. The clustering coefficient $c_{i}$ of each node *i* is the ratio between the number $e_{i}$ of edges that actually exist in all $k_{i}$ nearest neighbors and the number $k_{i}\left(k_{i}-1\right)/2$ of all possible edges between them, and it is expressed as $c_{i}=2e_{i}/k_{i}\left(k_{i}-1\right)$. The whole clustering coefficient $\bar{C}$ of the network is the average value of $c_{i}$ over all nodes in the network, which can be written as $\bar{C}=\sum_{i\in V(G)}c_{i}/N_{t}$.

**Theorem** **3.** *For the network G(t) with $N_{t}$ nodes, the solution of whole clustering coefficient of network G(t) is*

(4) $$\bar{C}=\frac{2\times 5^{t}-10\times 3^{t-2}}{3^{2t-2}\left(5^{t}+3\right)}\to 0,\;\mathrm{when}\;\;t\to \infty .$$

**Proof.**  Since the clustering coefficient of each node with the same degree in G(t) is also the same, let n(k) be the number of nodes with degree *k*, and c(k) represents the clustering coefficient of each node with degree *k*. For each degree *k*, we can calculate the clustering coefficient c(k) and the corresponding number n(k) of nodes with degree *k*, as shown in Table 1, so the whole clustering coefficient $\bar{C}$ of network G(t) can be calculated as follows:

(5) $$\begin{array}{cc} \bar{C} & =\sum_{k}\frac{c(k)\times n(k)}{N_{t}}=\frac{2}{3^{t}}\times \frac{4}{5^{t}+3}+\frac{4}{5^{t}+3}\sum_{t_{i}=1}^{t}\frac{5^{t_{i}-1}}{3^{t-t_{i}}}\\ \; & =\frac{2\times 5^{t}-10\times 3^{t-2}}{3^{2t-2}\left(5^{t}+3\right)}, \end{array}$$

we have $\bar{C}\to 0$ when t→∞, and G(t) is not a highly clustered network. However, the clustering coefficient of the complete graph $K_{N_{t}}$ is equal to 1, and there is a significant difference between G(t) and $K_{N_{t}}$ on this topological parameter. □

### 2.4. Diameter

The diameter is defined as the maximum of the shortest distances between all pairs of nodes in network G(t), denoted as D(G(t)), and it is often used to characterize the longest communication delay in complex network.

**Theorem** **4.** *For t≥0, the diameter of the sparse network G(t) is D(G(t))=t+1.*

**Proof.**  When G(t) is a small network, we can easily enumerate its diameter, such as t=0, D(G(0))=1. At time step t=1, the distance between two new nodes is the longest, and the path through them must contain an old node, so D(G(1))=2. For t>1, we can find that the diameter refers to the distance between a pair of new nodes. For simplicity of description, we denote the newly generated node at time $t_{i}$ as $t_{i}$; therefore, we only need to consider the maximum value of the shortest distance between two nodes *t*. According to the structure of G(t), when t=2, the shortest path between new nodes must be the path $P_{2}=2\to 1\to 0\to 2$, which contains nodes generated at time step 0,1,2. For t≥3, the shortest path between two new nodes must be a path $P_{t}=t\to t-1\to t-2\to \cdots \to 3\to 1\to 0\to 2\to t$. Hence, D(G(t))=t+1 is true for t≥0, which shows that the diameter scales logarithmically with the network order. For a complete graph $K_{N_{t}}$ with the same number of nodes, it is well known that its diameter is equal to 1, which means that the diameter of G(t) is larger than that of $K_{N_{t}}$. □

## 3. Random Walks and Electrical Networks

In this section, we aim to show the closed-form solution for the mean hitting time of our networks. Firstly, we introduce several notions and lemmas about electrical networks and random walks, then we provide the relationships between the mean hitting time and Kirchhoff index. The electrical network corresponding to a graph G(t) can be constructed by replacing each edge in G(t) with a unit resistor, but we still denote the resulting electrical network as G(t). The effective resistance $\Omega_{ij}$ between any two distinct nodes i,j∈V(t) is defined as the potential difference between them when a unit current from *i* to *j* is kept; when i=j, we set $\Omega_{ij}=0$.

**Lemma** **1** ([20])**.** *For an electrical network* G(t) with $N_{t}$ *nodes, the sum of effective resistances between all pairs of adjacent nodes can be written as*

(6) $$\sum_{i<j,(i,j)\in E(t)}\Omega_{ij}=N_{t}-1.$$

**Lemma** **2** ([21])**.** *For any pair of distinct node i and j in an electrical network G(t), $k_{i}$ and N(i) represent the degree of node i and its neighbors set, then the degree and effective resistance satisfy the following relationship:*

(7) $$k_{i}\Omega_{ij}+\sum_{s\in N(i)}\left(\Omega_{is}-\Omega_{js}\right)=2.$$

According to [22], the regular Kirchhoff index K(G(t)) of a network G(t) is defined as the sum of the effective resistances of all pairs of disordered nodes in G(t):

(8) $$K\left(G\left(t\right)\right)=\sum_{i,j\in V(t)}\Omega_{ij}.$$

Taking into account the influence of degree on the Kirchhoff index, the M-Kirchhoff index $K^{*}\left(G\left(t\right)\right)$ and the A-Kirchhoff index $K^{+}\left(G\left(t\right)\right)$ have been proposed in [23,24], respectively, and they are interpreted by the formula as

(9) $$K^{*}\left(G\left(t\right)\right)=\sum_{i,j\in V(t)}\left(d_{i}d_{j}\right)\Omega_{ij},$$

and

(10) $$K^{+}\left(G\left(t\right)\right)=\sum_{i,j\in V(t)}\left(d_{i}+d_{j}\right)\Omega_{ij}.$$

The unbiased discrete time random walks means that the particle starting from the current location jumps to each of its neighboring nodes with equal probability at every time step [25]. The hitting time $T_{ij}$ of network G(t) is a key quantity pertaining to random walk, and it is defined as the expected time taken by a particle jumping to the ending node *j* from the starting node *i* for the first time. The mean hitting time $\bar{T}\left(G\left(t\right)\right)$ is the average of hitting times over all node pairs [26], and it can be solved by the K(G(t)) and the network order and size of G(t).

**Lemma** **3.** *For a network G(t) with $N_{t}$ nodes and $E_{t}$ edges, K(G(t)) represents its regular Kirchhoff index, then the mean hitting time $\bar{T}\left(G\left(t\right)\right)$ can be expressed as*

(11) $$\bar{T}\left(G\left(t\right)\right)=\frac{E_{t}\cdot K\left(G\left(t\right)\right)}{N_{t}\left(N_{t}-1\right)}.$$

**Proof.**  The Kirchhoff index K(G(t)) can be represented in terms of the $N_{t}-1$ non-zero eigenvalues of the Laplacian matrix $L_{t}$ for network G(t) as $K\left(G\left(t\right)\right)=2N_{t}\sum_{i=2}^{N_{t}}\frac{1}{\lambda_{i}}$[24]. The mean hitting time $\bar{T}\left(G\left(t\right)\right)$ of network G(t) is the average of hitting times over all $N_{t}\left(N_{t}-1\right)$ node pairs, and it is expressed as $\bar{T}\left(G\left(t\right)\right)=\frac{1}{N_{t}\left(N_{t}-1\right)}\sum_{i=1,i\neq j}^{N_{t}}\sum_{j=1}^{N_{t}}H_{ij}$, where $H_{ij}$ is the hitting time from node *i* to another node *j*. In addition, $\bar{T}\left(G\left(t\right)\right)$ can be expressed in terms of the non-zero eigenvalues of the Laplacian matrix $L_{t}$[18], that is $\bar{T}\left(G\left(t\right)\right)=\frac{2E_{t}}{N_{t}\left(N_{t}-1\right)}\sum_{i=2}^{N_{t}}\frac{1}{\lambda_{i}}$, then we can obtain the equation $\bar{T}\left(G\left(t\right)\right)=\frac{1}{N_{t}\left(N_{t}-1\right)}E_{t}\cdot K\left(G\left(t\right)\right)$ by combining the above equations. □

### 3.1. Related Matrices

All the nodes of a given network G(t) are marked as $1,2,3,\cdots ,N_{t}$, respectively, and the adjacency relations between all nodes and edges are implicit in an adjacency matrix $A_{t}={\left(a_{ij}\right)}_{N_{t}\times N_{t}}$, where $a_{ij}=1$ if node *i* and *j* are connected by an edge in the network, and $a_{ij}=0$ if there is no edge between *i* and *j*. Let $D_{t}$ be the diagonal degree matrix of G(t). Its *i*-th diagonal entry is the degree $k_{i}^{\left(t\right)}$ of node *i*, and the remaining entries are zero. The Laplacian matrix of G(t) is defined as $L_{t}=D_{t}-A_{t}$.

Use α=V(t−1) and $\beta =\bar{V}\left(t\right)$ to abbreviate the old node set and the new node set in network G(t+1), and the number of new nodes is $|\bar{V}\left(t+1\right)|=2\times 5^{t}$. The network G(t+1) is generated iteratively by G(t), then we show the recursive relationship between two consecutive time steps of these matrices. The adjacency matrix $A_{t+1}$ can be written in block form as

(12) $$A_{t+1}=\left(\begin{array}{cc} A_{t+1}^{\alpha ,\alpha } & A_{t+1}^{\alpha ,\beta }\\ A_{t+1}^{\beta ,\alpha } & A_{t+1}^{\beta ,\beta } \end{array}\right)=\left(\begin{array}{cc} A_{t} & A_{t+1}^{\alpha ,\beta }\\ A_{t+1}^{\beta ,\alpha } & 0 \end{array}\right),$$

where $A_{t+1}^{\beta ,\alpha }={\left(A_{t+1}^{\alpha ,\beta }\right)}^{T}$ is obvious according to the definition of adjacency matrix, and $A_{t+1}^{\beta ,\beta }$ is the zero matrix with order $|\bar{V}\left(t+1\right)|\times |\bar{V}\left(t+1\right)|$. On the other hand, the diagonal matrix $D_{t+1}$ satisfies

(13) $$D_{t+1}=\left(\begin{array}{cc} D_{t+1}^{\alpha ,\alpha } & D_{t+1}^{\alpha ,\beta }\\ 0 & D_{t+1}^{\beta ,\beta } \end{array}\right)=\left(\begin{array}{cc} 3D_{t} & 0\\ 0 & 2I \end{array}\right),$$

where the symbol *I* represents the identity matrix with order $|\bar{V}\left(t+1\right)|\times |\bar{V}\left(t+1\right)|$. Equation (13) is based on the fact that the nodes contained in set β are 2 degree nodes, the degree of every node in set α increases by a factor 3. Thus, the Laplacian matrix $L_{t+1}$ of network G(t+1) can be expressed as

(14) $$L_{t+1}=\left(\begin{array}{cc} 3D_{t}-A_{t} & -A_{t+1}^{\alpha ,\beta }\\ -A_{t+1}^{\beta ,\alpha } & 2I \end{array}\right).$$

**Theorem** **5.** *For the sparse network G(t+1) after t+1 time steps, we have $A_{t+1}^{\alpha ,\beta }A_{t+1}^{\beta ,\alpha }=2D_{t}+2A_{t}$.*

**Proof.**  The left side and right side of equation $A_{t+1}^{\alpha ,\beta }A_{t+1}^{\beta ,\alpha }=2D_{t}+2A_{t}$ are denoted by ${\bar{M}}_{t}$ and $M_{t}$, respectively; thus, the entries of $M_{t}$ are

$$M_{t}\left(i,j\right)=\left\{\begin{array}{cc} 2k_{i}^{\left(t\right)}, & i=j;\\ 2A_{t}\left(i,j\right), & i\neq j. \end{array}\right.$$

□

Our main task is to verify that the entries ${\bar{M}}_{t}\left(i,j\right)$ of ${\bar{M}}_{t}$ are equivalent to those of $M_{t}$. Matrix $A_{t+1}^{\beta ,\alpha }$ can be partitioned into $N_{t}$ column vectors $x_{i}={\left(x_{i,N_{t}+1},x_{i,N_{t}+2},\cdots ,x_{i,N_{t+1}}\right)}^{T}$ for $i=1,2,\cdots ,N_{t}$, that is $A_{t+1}^{\beta ,\alpha }=\left(x_{1},x_{2},\cdots ,x_{N_{t}}\right)$, and we have $A_{t+1}^{\alpha ,\beta }={\left(x_{1},x_{2},\cdots ,x_{N_{t}}\right)}^{T}$ due to $A_{t+1}^{\alpha ,\beta }={\left(A_{t+1}^{\beta ,\alpha }\right)}^{T}$, and we can calculate the product of the two matrices as $A_{t+1}^{\alpha ,\beta }A_{t+1}^{\beta ,\alpha }={\left(x_{i}^{T}x_{j}\right)}_{N_{t}\times N_{t}}$.

The entries ${\bar{M}}_{t}\left(i,j\right)$ of ${\bar{M}}_{t}$ can be determined by distinguishing two cases. (a) When i=j, the diagonal entry is ${\bar{M}}_{t}\left(i,i\right)=x_{i}^{T}x_{i}$, and we can obtain ${\bar{M}}_{t}\left(i,i\right)=2k_{i}^{\left(t\right)}=M_{t}\left(i,i\right)$. (b) When i≠j, the non-diagonal entry of matrix ${\bar{M}}_{t}$ is equal to

(15) $$\begin{array}{cc} {\bar{M}}_{t}\left(i,j\right) & =x_{i}^{T}x_{j}=\sum_{s\in \beta }\left(x_{i,s}x_{j,s}\right)=\sum_{\begin{array}{c} A_{t+1}\left(i,s\right)=1\\ A_{t+1}\left(j,s\right)=1 \end{array}}A_{t}\left(i,j\right)\\ \; & =2A_{t}\left(i,j\right)=M_{t}\left(i,j\right). \end{array}$$

Before going on, we introduce a concept about {1}-inverse of a matrix [27]. Matrix *M* is called a {1}-inverse of *X* if XMX=X holds, let $X^{†}$ be one of the {1}-inverses of *X*. A lemma about the {1}-inverse of a block matrix is shown below.

**Lemma** **4** ([18])**.** *Let matrix $P=\left(\begin{array}{cc} X & Y\\ Y^{T} & Z \end{array}\right)$ be a block matrix, and Z is nonsingular if there exists a {1}-inverse $D^{†}$ for $D=X-YZ^{-1}Y^{T}$, then the {1}-inverse of matrix P is a matrix $P^{†}=\left(\begin{array}{cc} D^{†} & -D^{†}YZ^{-1}\\ -Z^{-1}Y^{T}D^{†} & Z^{-1}Y^{T}D^{†}YZ^{-1}+Z^{-1} \end{array}\right)$.*

### 3.2. Effective Resistances

For a connected network G(t), the effective resistance $\Omega_{ij}^{\left(t\right)}$ between any pair of nodes can be obtained from the elements of any {1}-inverse of its Laplacian matrix, and we can refer to the following lemma.

**Lemma** **5** ([28])**.** *For a given G(t), let $L_{ij}^{†}$ be the (i,j)-th element of any {1}-inverse $L_{t}^{†}$ of its Laplacian matrix $L_{t}$. For any two nodes i,j∈V(t), the effective resistance $\Omega_{ij}^{\left(t\right)}$ can be expressed in terms of the elements of $L_{t}^{†}$ as $\Omega_{ij}^{\left(t\right)}=L_{ii}^{†}+L_{jj}^{†}-L_{ij}^{†}-L_{ji}^{†}$.*

Next, we show that the effective resistance between any two nodes in G(t+1) can be represented in terms of effective resistances of node pairs in G(t). In the following calculation process, we divide the nodes into the old node and the new node to investigate the effective resistance between them. To achieve this goal, we introduce some variables and define $\Omega_{X,Y}^{\left(t\right)}=\sum_{i\in X,j\in Y}\Omega_{ij}^{\left(t\right)}$ for any two subsets *X* and *Y* of set V(t) on G(t). Then, for a node $i\in \bar{V}\left(t+1\right)$ in G(t+1), we define $\Omega_{\Delta_{i}}^{\left(t\right)}=\Omega_{ab}^{\left(t+1\right)}$, where $\Delta_{i}=\left\{a,b\right\}$ is the neighbors set of node *i* and a,b∈V(t).

**Lemma** **6.** *For the effective resistance between the node pairs in the network G(t+1), the following propositions are established for t≥0,*

*(1) Let i,j∈V(t) be a pair of old nodes in G(t+1), then $\Omega_{ij}^{\left(t+1\right)}$ obeys the relation*

(16) $$\Omega_{ij}^{\left(t+1\right)}=\frac{1}{2}\Omega_{ij}^{\left(t\right)}.$$

*(2) Let $i\in \bar{V}\left(t+1\right)$ be a new node in network G(t), then*

(17) $$\Omega_{i,\Delta_{i}}^{\left(t+1\right)}=1+\frac{1}{2}\Omega_{\Delta_{i}}^{\left(t+1\right)}.$$

*(3) Let $i\in \bar{V}\left(t+1\right)$ and j∈V(t) be a new node and an old node in network G(t+1), respectively, then the following equation holds*

(18) $$\Omega_{ij}^{\left(t+1\right)}=\frac{1}{2}\left(1-\frac{1}{2}\Omega_{\Delta_{i}}^{\left(t+1\right)}+\Omega_{j,\Delta_{i}}^{\left(t+1\right)}\right).$$

*(4) Let $i,j\in \bar{V}\left(t+1\right)$ be a pair of distinct new nodes in network G(t+1), then $\Omega_{ij}^{\left(t+1\right)}$ obeys*

(19) $$\Omega_{ij}^{\left(t+1\right)}=1-\frac{1}{4}\left(\Omega_{\Delta_{i}}^{\left(t+1\right)}+\Omega_{\Delta_{j}}^{\left(t+1\right)}\right)+\frac{1}{4}\Omega_{\Delta_{i},\Delta_{j}}.$$

The detailed proof of Lemma 6 is given in Appendix A.

## 4. Mean Hitting Time

Based on the above preparations, we determine the mean hitting time for network G(t) using the connection between the mean hitting time and the Kirchhoff index. Firstly, we calculate the exact solutions of three auxiliary variables, including $K_{X,Y}\left(t\right)=\sum_{i\in X,j\in Y}\Omega_{ij}^{\left(t\right)}$, $K_{X,Y}^{*}\left(t\right)=\sum_{i\in X,j\in Y}k_{i}^{\left(t\right)}k_{j}^{\left(t\right)}\Omega_{ij}^{\left(t\right)}$, and $K_{X,Y}^{+}\left(t\right)=\sum_{i\in X,j\in Y}\left(k_{i}^{\left(t\right)}+k_{j}^{\left(t\right)}\right)\Omega_{ij}^{\left(t\right)}$ for two subsets *X* and *Y* of set V(t) in network G(t), and give the relationships between them. The following lemmas can support our main result.

**Lemma** **7.** *For network G(t+1), $i\in \bar{V}\left(t+1\right)$ is a new node and j∈V(t) is an old node, Y⊆V(t), $\Delta_{i}$ is the set of all neighbors of node i, then the following two summation formulas hold:*

*(a) $\sum_{i\in \bar{V}\left(t+1\right)}\Omega_{\Delta_{i}}^{\left(t+1\right)}=N_{t}-1$.*

*(b) $\sum_{i\in \bar{V}\left(t+1\right)}\Omega_{\Delta_{i},Y}^{\left(t+1\right)}=2k_{j}^{\left(t\right)}\sum_{j\in V(t)}\Omega_{j,Y}^{\left(t+1\right)}$.*

**Proof.**  (a) Since each edge of network G(t) can generate two new nodes of network G(t+1), then we have

(20) $$\begin{array}{cc} \sum_{i\in \bar{V}\left(t+1\right)}\Omega_{\Delta_{i}}^{\left(t+1\right)} & =2\sum_{\left(s,t)\in E(t\right)}\Omega_{s,t}^{\left(t+1\right)}=2\sum_{\left(s,t)\in E(t\right)}\frac{1}{2}\Omega_{s,t}^{\left(t\right)}\\ \; & =N_{t}-1. \end{array}$$

(b) For any old node j∈V(t), there are $k_{j}^{\left(t+1\right)}-k_{j}^{\left(t\right)}=2k_{j}^{\left(t\right)}$ new nodes in $\bar{V}\left(t+1\right)$ that are adjacent to *j*, so $\Omega_{j,Y}^{\left(t+1\right)}$ is summed $2k_{j}^{\left(t\right)}$ times. □

**Lemma** **8.** *The M-Kirchhoff index and the A-Kirchhoff index of our network G(t) are equal to*

(21) $$K_{V_{t},V_{t}}^{*}\left(t\right)=-\frac{38}{15}{\left(\frac{25}{2}\right)}^{t}+\frac{14}{5}\times 5^{2t}+\frac{26}{15}\times 5^{t},$$

*and*

(22) $$K_{V_{t},V_{t}}^{+}\left(t\right)=\frac{19}{9}{\left(\frac{5}{2}\right)}^{t}-\frac{19}{15}{\left(\frac{25}{2}\right)}^{t}+\frac{161}{90}\times 5^{2t}+\frac{13}{15}\times 5^{t}+\frac{1}{2}.$$

The proof of Lemma 8 is given in Appendix B.

**Theorem** **6.** *The solution of regular Kirchhoff index of our network G(t) is*

(23) $$\begin{array}{cc} K_{V_{t},V_{t}}\left(t\right) & =\frac{95}{72}{\left(\frac{1}{2}\right)}^{t}+\frac{19}{36}{\left(\frac{5}{2}\right)}^{t}-\frac{19}{120}{\left(\frac{25}{2}\right)}^{t}+\frac{49}{180}\times 5^{2t}+\frac{13}{45}\times 5^{t}-\frac{1}{4}. \end{array}$$

**Proof.**  Through the two types of Kirchhoff indexes obtained by Lemma 8, we deduce the relationship between the regular Kirchhoff index and them. Divide all the nodes in the network G(t+1) into new node and old node, then $K_{V_{t+1},V_{t+1}}\left(t+1\right)$ is equal to

(24) $$\begin{array}{cc} \; & K_{\alpha ,\alpha }\left(t+1\right)+2K_{\alpha ,\beta }\left(t+1\right)+K_{\beta ,\beta }\left(t+1\right)\\ = & \frac{1}{2}K_{V_{t},V_{t}}\left(t\right)+2K_{\alpha ,\beta }\left(t+1\right)+\frac{1}{4}K_{\beta ,\beta }^{*}\left(t+1\right)\\ = & \frac{1}{2}K_{V_{t},V_{t}}\left(t\right)+\frac{1}{2}K_{V_{t},V_{t}}^{+}\left(t\right)+\frac{1}{2}K_{V_{t},V_{t}}^{*}\left(t\right)+\frac{35}{8}\times 5^{2t}-\frac{3}{8}.\\ = & \frac{1}{2}K_{V_{t},V_{t}}\left(t\right)+\frac{19}{18}{\left(\frac{5}{2}\right)}^{t}-\frac{19}{10}{\left(\frac{25}{2}\right)}^{t}+\frac{2401}{360}\times 5^{2t}+\frac{13}{10}\times 5^{t}-\frac{1}{8}. \end{array}$$

□

**Figure 2:**
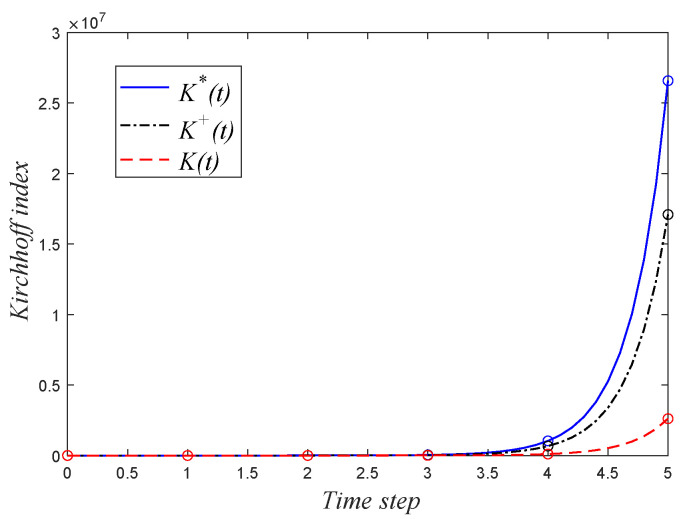
The schematic diagram of the three types of Kirchhoff indexes.

Considering $K_{V_{0},V_{0}}\left(0\right)=2$, plugging Equations (21) and (22) into Equation (24) yields the solution of Kirchhoff index of network G(t), as shown in Equation (23). Figure 2 shows a schematic diagram of the three types of Kirchhoff indexes of network G(t). We are ready to show the result for the mean hitting time $\bar{T}\left(G\left(t\right)\right)$ of G(t).

**Theorem** **7.** *For t≥0, the closed-form solution for the mean hitting time of network G(t) is*

(25) $$\begin{array}{cc} \bar{T}\left(G\left(t\right)\right) & =\frac{1}{5^{2t}+4\times 5^{t}+3}[\frac{95}{18}{\left(\frac{5}{2}\right)}^{t}+\frac{19}{9}{\left(\frac{25}{2}\right)}^{t}-\frac{19}{30}{\left(\frac{125}{2}\right)}^{t}\\ \; & \;\;\;+\frac{49}{45}5^{3t}+\frac{52}{45}5^{2t}-5^{t}], \end{array}$$

*then $\bar{T}\left(G_{t}\right)\approx N_{t}$ for t→∞.*

**Proof.**  Since Lemma 3 and the total number of edges of whole network is $E_{t}=5^{t}=2N_{t}-3$, we have

(26) $$\bar{T}\left(G\left(t\right)\right)=\frac{2N_{t}-3}{N_{t}\left(N_{t}-1\right)}K_{V_{t},V_{t}}.$$

It is easy to obtain the result in Equation (25) by substituting Equation (23) into Equation (26). We continue to express $\bar{T}\left(G\left(t\right)\right)$ as a function of the network order $N_{t}$, it can be observed that $t=\frac{\ln(2N_{t}-3)}{\ln5}$ from the exact value of $N_{t}$; hence, the mean hitting time $\bar{T}\left(G\left(t\right)\right)$ of G(t) can be expressed in terms of network order as

(27) $$\begin{array}{cc} \bar{T}\left(G\left(t\right)\right) & =\frac{2N_{t}-3}{N_{t}\left(N_{t}-1\right)}[\frac{95}{72}{\left(2N_{t}-3\right)}^{-\frac{\ln2}{\ln5}}+\frac{19}{36}{\left(2N_{t}-3\right)}^{\frac{\ln5-\ln2}{\ln5}}\\ \; & \;\;\;-\frac{19}{120}{\left(2N_{t}-3\right)}^{\frac{\ln25-\ln2}{\ln5}}+\frac{49}{180}{\left(2N_{t}-3\right)}^{2}+\frac{13}{45}\left(2N_{t}-3\right)-\frac{1}{4}]. \end{array}$$

Therefore, when t→∞, for a large network, we have

(28) $$\bar{T}\left(G\left(t\right)\right)\approx \frac{98}{45}N_{t},$$

which shows $\bar{T}\left(G\left(t\right)\right)$ increases linearly with the total number of nodes in our network; the mean hitting time of the random walks shown is similar to that of the complete graph; and they all have high transmission efficiency. □

## 5. Conclusions

In this paper, we have presented a class of a sparse network G(t) and have pointed out the differences between G(t) and the complete graph $K_{N_{t}}$ with the same order in several topological characteristics. The main differences are G(t) has a scale-free property, while $K_{N_{t}}$ does not. The scale-free feature is a shock discovery in real complex systems. $K_{N_{t}}$ is not sparse, but G(t) is sparse; it is rare to achieve a tight connections like a complete graph in a real network. It has been proven that the mean hitting time of the complete graph is minimal and increases linearly with the network order. Based on the relationship between the mean hitting time and the Kirchhoff index, we have calculated a closed-form solution to the mean hitting time of our network, and the result shows that the dominant scaling of which exhibits the same behavior as that of a complete graph. We hope that our work will be instructive for the design and construction of complex networks with efficient navigation.

## Figures and Tables

**Table 1:** The degree and clustering coefficient of node in G(t).

*t*	*k*	n(k)	c(k)
0	$3^{t}$	2	$2/3^{t}$
1	$2\times 3^{t-1}$	$2\times 5^{0}$	$1/3^{t-1}$
2	$2\times 3^{t-2}$	$2\times 5^{1}$	$1/3^{t-2}$
⋯	⋯	⋯	⋯
$t_{i}$	$2\times 3^{t-t_{i}}$	$2\times 5^{t_{i}-1}$	$1/3^{t-t_{i}}$
⋯	⋯	⋯	⋯
t−1	$2\times 3^{1}$	$2\times 5^{t-2}$	$1/3^{1}$
*t*	$2\times 3^{0}$	$2\times 5^{t-1}$	$1/3^{0}$

## Data Availability

Not applicable.

## Appendix A. The Proof of Lemma 6

In Appendix A, we give the proofs of the four propositions in Lemma 6.

(1) The nodes are divided into two parts; the new node set is abbreviated as β, and the old node set is denoted as α, then any {1}-inverse $L_{t+1}^{†}$ of Laplacian matrix $L_{t+1}$ can be written as the form of the block matrix

(A1) $$L_{t+1}^{†}=\left(\begin{array}{cc} L_{\alpha ,\alpha }^{†} & L_{\alpha ,\beta }^{†}\\ L_{\beta ,\alpha }^{†} & L_{\beta ,\beta }^{†} \end{array}\right).$$

By Equation (14) and Lemma 4, applying the conditions in Theorem 5, we can clearly obtain the relationship between block $L_{\alpha ,\alpha }^{†}$ of matrix $L_{t+1}^{†}$ and matrix $L_{t}^{†}$,

(A2) $$\begin{array}{cc} L_{\alpha ,\alpha }^{†} & ={\left(3D_{t}-A_{t}-\left(-A_{t+1}^{\alpha ,\beta }\right){\left(2I\right)}^{-1}\left(-A_{t+1}^{\beta ,\alpha }\right)\right)}^{†}\\ \; & ={\left(3D_{t}-A_{t}-\frac{1}{2}\left(2D_{t}+2A_{t}\right)\right)}^{†}=\frac{1}{2}L_{t}^{†}. \end{array}$$

According to Lemma 5 and Equation (A2), for i,j∈V(t), we reveal the relationship between the effective resistance at two consecutive iterations for any pair of old nodes in network G(t+1),

(A3) $$\begin{array}{cc} \Omega_{ij}^{\left(t+1\right)} & =L_{\alpha ,\alpha }^{†}\left(i,i\right)+L_{\alpha ,\alpha }^{†}\left(j,j\right)-L_{\alpha ,\alpha }^{†}\left(i,j\right)-L_{\alpha ,\alpha }^{†}\left(j,i\right)\\ \; & =\frac{1}{2}\left(L_{t}^{†}\left(i,i\right)+L_{t}^{†}\left(j,j\right)-L_{t}^{†}\left(i,j\right)-L_{t}^{†}\left(j,i\right)\right)\\ \; & =\frac{1}{2}\Omega_{ij}^{\left(t\right)}. \end{array}$$

(2) For $i\in \bar{V}\left(t+1\right)$ and its neighboring node set $\Delta_{i}=\left\{a,b\right\}$, we can obtain the following two equations $2\Omega_{ia}^{\left(t+1\right)}+\Omega_{i,\Delta_{i}}^{\left(t+1\right)}-\Omega_{a,\Delta_{i}}^{\left(t+1\right)}=2$ and $2\Omega_{ib}^{\left(t+1\right)}+\Omega_{i,\Delta_{i}}^{\left(t+1\right)}-\Omega_{b,\Delta_{i}}^{\left(t+1\right)}=2$ by applying Lemma 2 to the two old neighboring nodes of node *i*. Then, we add the results of these two equations together to give

(A4) $$2\Omega_{i,\Delta_{i}}^{\left(t+1\right)}+2\Omega_{i,\Delta_{i}}^{\left(t+1\right)}-\Omega_{\Delta_{i},\Delta_{i}}^{\left(t+1\right)}=4,$$

the final result of the above equation simplification is shown as

(A5) $$\Omega_{i,\Delta_{i}}^{\left(t+1\right)}=1+\frac{1}{4}\Omega_{\Delta_{i},\Delta_{i}}^{\left(t+1\right)}=1+\frac{1}{2}\Omega_{\Delta_{i}}^{\left(t+1\right)}.$$

(3) For a given new node $i\in \bar{V}\left(t+1\right)$ and an old node j∈V(t), we also have $d_{i}^{\left(t+1\right)}\Omega_{ij}^{\left(t+1\right)}+\Omega_{i,\Delta_{i}}^{\left(t+1\right)}-\Omega_{j,\Delta_{i}}^{\left(t+1\right)}=2$ with the help of Lemma 2, and considering $k_{i}^{\left(t+1\right)}=2$ and proposition (2), we have

(A6) $$\Omega_{ij}^{\left(t+1\right)}=\frac{1}{2}\left(1-\frac{1}{2}\Omega_{\Delta_{i}}^{\left(t+1\right)}+\Omega_{j,\Delta_{i}}^{\left(t+1\right)}\right).$$

(4) For two new nodes $i,j\in \bar{V}\left(t+1\right)$, i≠j, we can write $d_{i}^{\left(t+1\right)}\Omega_{ij}^{\left(t+1\right)}+\Omega_{i,\Delta_{i}}^{\left(t+1\right)}-\Omega_{j,\Delta_{i}}^{\left(t+1\right)}=2$ with the support of the Lemma 2, combining condition $k_{i}^{\left(t+1\right)}=2$ and Propositions (2) and (3), we obtain the following derivation process,

(A7) $$\begin{array}{cc} \Omega_{ij}^{\left(t+1\right)} & =\frac{1}{2}\left(2-\Omega_{i,\Delta_{i}}^{\left(t+1\right)}+\Omega_{j,\Delta_{i}}^{\left(t+1\right)}\right)=\frac{1}{2}\left(2-\Omega_{i,\Delta_{i}}^{\left(t+1\right)}+\sum_{s\in \Delta_{i}}\Omega_{j,s}^{\left(t+1\right)}\right)\\ \; & =\frac{1}{2}\left[1-\frac{1}{2}\Omega_{\Delta_{i}}^{\left(t+1\right)}+\sum_{s\in \Delta_{i}}\frac{1}{2}\left(1-\frac{1}{2}\Omega_{\Delta_{j}}^{\left(t+1\right)}+\Omega_{k,\Delta_{j}}^{\left(t+1\right)}\right)\right]\\ \; & =1-\frac{1}{4}\left(\Omega_{\Delta_{i}}^{\left(t+1\right)}+\Omega_{\Delta_{j}}^{\left(t+1\right)}\right)+\frac{1}{4}\Omega_{\Delta_{i},\Delta_{j}}^{\left(t+1\right)}. \end{array}$$

As shown above, the four propositions in the Lemma 6 have been proved.

## Appendix B. The Proof of Lemma 8

As defined above, α and β are the old node set and new node set in network G(t+1), the M-Kirchhoff index can be calculated by $K_{V_{t+1},V_{t+1}}^{*}\left(t+1\right)=K_{\alpha ,\alpha }^{*}\left(t+1\right)+K_{\alpha ,\beta }^{*}\left(t+1\right)+K_{\beta ,\alpha }^{*}\left(t+1\right)+K_{\beta ,\beta }^{*}\left(t+1\right)$. Because $K_{\alpha ,\beta }^{*}\left(t+1\right)$ and $K_{\beta ,\alpha }^{*}\left(t+1\right)$ are equal to each other, we only evaluate the three terms other than $K_{\beta ,\alpha }^{*}\left(t+1\right)$ on the right side of above equation. According to Lemma 6 (1), we can write the first term as

(A8) $$\begin{array}{cc} K_{\alpha ,\alpha }^{*}\left(t+1\right) & =\sum_{i\in V(t),j\in V(t)}k_{i}^{\left(t+1\right)}k_{j}^{\left(t+1\right)}\Omega_{i,j}^{\left(t+1\right)}\\ \; & =3^{2}\times \frac{1}{2}\sum_{i\in V(t),j\in V(t)}k_{i}^{\left(t\right)}k_{j}^{\left(t\right)}\Omega_{i,j}^{\left(t\right)}=\frac{9}{2}K_{V_{t},V_{t}}^{*}\left(t\right). \end{array}$$

Refer to the expression of the effective resistance between the new node and old node given in Lemma 6 (3), and the second term can be investigated by a similar derivation method,

(A9) $$\begin{array}{cc} K_{\alpha ,\beta }^{*}\left(t+1\right) & =\sum_{i\in \bar{V}\left(t+1\right),j\in V\left(t\right)}k_{i}^{\left(t+1\right)}k_{j}^{\left(t+1\right)}\Omega_{i,j}^{\left(t+1\right)}\\ \; & =\sum_{i\in \bar{V}\left(t+1\right),j\in V\left(t\right)}2\left(3k_{j}^{\left(t\right)}\right)\frac{1}{2}\left(1-\frac{1}{2}\Omega_{\Delta_{i}}^{\left(t+1\right)}+\Omega_{\Delta_{i},j}^{\left(t+1\right)}\right)\\ \; & =\sum_{i\in \bar{V}\left(t+1\right)}\left(1-\frac{1}{2}\Omega_{\Delta_{i}}^{\left(t+1\right)}\right)\left(\sum_{j\in V(t)}3k_{j}^{\left(t\right)}\right)\\ \; & +3\sum_{i\in \bar{V}\left(t+1\right),j\in V\left(t\right)}k_{j}^{\left(t\right)}\Omega_{\Delta_{i},j}^{\left(t+1\right)}, \end{array}$$

for a node $i\in \bar{V}\left(t+1\right)$, we note that $k_{i}^{\left(t+1\right)}=2$ and the transformation equation given by Lemma 7, and Equation (A9) is reformulated as

(A10) $$\begin{array}{cc} K_{\alpha ,\beta }^{*}\left(t+1\right) & =3\times 2\times E_{t}\left(\right|\bar{V}\left(t+1\right)\left|-\frac{1}{2}\left(N_{t}-1\right)\right)\\ \; & \;\;\;\;\;+3\cdot 2\sum_{i,j\in V(t)}k_{i}^{\left(t\right)}k_{j}^{\left(t\right)}\Omega_{i,j}^{\left(t+1\right)}\\ \; & =3K_{V_{t},V_{t}}^{*}\left(t\right)+\frac{21}{2}\cdot 5^{2t}-\frac{3}{2}\cdot 5^{t}. \end{array}$$

Considering the effective resistance between the new node, we need to calculate the last term $K_{\beta ,\beta }^{*}\left(t+1\right)$ by Lemma 6 (4), then $K_{\beta ,\beta }^{*}\left(t+1\right)$ is equal to

(A11) $$\begin{array}{cc} \; & \;\;\;\sum_{i,j\in \bar{V}\left(t+1\right),i\neq j}d_{i}^{\left(t+1\right)}d_{j}^{\left(t+1\right)}\Omega_{i,j}^{\left(t+1\right)}\\ \; & =4\sum_{i,j\in \bar{V}\left(t+1\right),i\neq j}\left[1-\frac{1}{4}\left(\Omega_{\Delta_{i}}^{\left(t+1\right)}+\Omega_{\Delta_{j}}^{\left(t+1\right)}\right)+\frac{1}{4}\Omega_{\Delta_{i},\Delta_{j}}^{\left(t+1\right)}\right]\\ \; & =4|\bar{V}\left(t+1\right)|(|\bar{V}\left(t+1\right)|-1)-2(|\bar{V}\left(t+1\right)\left|-1\right)\sum_{i\in \bar{V}\left(t+1\right)}\Omega_{\Delta_{i}}^{\left(t+1\right)}\\ \; & +(\sum_{i,j\in \bar{V}\left(t+1\right)}\Omega_{\Delta_{i},\Delta_{j}}^{\left(t+1\right)}-\sum_{i\in \bar{V}\left(t+1\right)}\Omega_{\Delta_{i},\Delta_{i}}^{\left(t+1\right)}). \end{array}$$

At the same time, according to the definition of equation $\Omega_{X,Y}^{\left(t\right)}=\sum_{i\in X,j\in Y}\Omega_{ij}^{\left(t\right)}$, the equation $\sum_{i\in \bar{V}\left(t+1\right)}\Omega_{\Delta_{i},\Delta_{i}}^{\left(t+1\right)}=2\sum_{i\in \bar{V}\left(t+1\right)}\Omega_{\Delta_{i}}^{\left(t+1\right)}$ can be easily obtained. Refer to the Lemma 7, we obtain

(A12) $$\begin{array}{cc} K_{\beta ,\beta }^{*}\left(t+1\right) & =4|\bar{V}\left(t+1\right)|(|\bar{V}\left(t+1\right)|-1)-2\left(N_{t}-1\right)\left|\bar{V}\left(t+1\right)\right|\\ \; & \;\;\;\;\;+4\sum_{x,y\in V_{t}}d_{x}^{\left(t\right)}d_{y}^{\left(t\right)}\Omega_{x,y}^{\left(t+1\right)}\\ \; & =2K_{V_{t},V_{t}}^{*}\left(t\right)+4|\bar{V}\left(t+1\right)|(|\bar{V}\left(t+1\right)\left|-1\right)\\ \; & \;\;\;\;\;-2|\bar{V}\left(t+1\right)|\left(N_{t}-1\right). \end{array}$$

Taking Equation (A12) into consideration and inserting the results of the three equations Equations (A8), (A10) and (A12) into $K_{V_{t+1},V_{t+1}}^{*}\left(t+1\right)=K_{\alpha ,\alpha }^{*}\left(t+1\right)+K_{\alpha ,\beta }^{*}\left(t+1\right)+K_{\beta ,\alpha }^{*}\left(t+1\right)+K_{\beta ,\beta }^{*}\left(t+1\right)$ yields

(A13) $$\begin{array}{cc} K_{V_{t+1},V_{t+1}}^{*}\left(t+1\right) & =\frac{25}{2}K_{V_{t},V_{t}}^{*}\left(t\right)+35\times 5^{2t}-13\times 5^{t}, \end{array}$$

the above calculation process shows a recursive formula about $K_{V_{t+1},V_{t+1}}^{*}\left(t+1\right)$. By adding a simple condition $K_{V_{0},V_{0}}^{*}\left(0\right)=2$, we obtain the desired result about $K_{V_{t},V_{t}}^{*}\left(t\right)$, as shown in Equation (21).

On the other hand, we consider the A-Kirchhoff index of network, and we also have the following recursive relation governing $K_{V_{t+1},V_{t+1}}^{+}\left(t+1\right)$ and $K_{V_{t},V_{t}}^{+}\left(t\right)$. Obviously, $K_{V_{t+1},V_{t+1}}^{+}\left(t+1\right)=K_{\alpha ,\alpha }^{+}\left(t+1\right)+2K_{\alpha ,\beta }^{+}\left(t+1\right)+K_{\beta ,\beta }^{+}\left(t+1\right)$, so we can deduce the following relations for the three terms of this equation,

(A14) $$\begin{array}{cc} K_{\alpha ,\alpha }^{+}\left(t+1\right) & =\frac{3}{2}K_{\alpha ,\alpha }^{+}\left(t\right),\;\;K_{\beta ,\beta }^{+}\left(t+1\right)=K_{\beta ,\beta }^{*}\left(t+1\right)\\ 2K_{\alpha ,\beta }^{+}\left(t+1\right) & =2\sum_{i\in \bar{V}\left(t+1\right),j\in V\left(t\right)}\left(2+k_{j}^{\left(t+1\right)}\right)\Omega_{i,j}^{\left(t+1\right)}\\ \; & =4K_{\alpha ,\beta }\left(t+1\right)+K_{\alpha ,\beta }^{*}\left(t+1\right). \end{array}$$

We have calculated the precise values of $K_{\beta ,\beta }^{*}\left(t+1\right)$ and $K_{\alpha ,\beta }^{*}\left(t+1\right)$ above, so the focus of the following calculation is to calculate $K_{\alpha ,\beta }\left(t+1\right)$. By using Lemma 6 (3), we have

(A15) $$\begin{array}{cc} K_{\alpha ,\beta }\left(t+1\right) & =\sum_{i\in \bar{V}\left(t+1\right),j\in V\left(t\right)}\frac{1}{2}\left(1-\frac{1}{2}\Omega_{\Delta_{i}}^{\left(t+1\right)}+\Omega_{j,\Delta_{i}}^{\left(t+1\right)}\right)\\ \; & =\sum_{i\in \bar{V}\left(t+1\right),j\in V\left(t\right)}\frac{1}{2}\left(1-\frac{1}{2}\Omega_{\Delta_{i}}^{\left(t+1\right)}\right)+\frac{1}{2}\sum_{i,j\in V(t)}k_{i}^{\left(t\right)}\Omega_{i,j}^{\left(t\right)}\\ \; & =\frac{1}{4}K_{V_{t},V_{t}}^{+}\left(t\right)+\frac{1}{2}N_{t}\left(\right|\bar{V}\left(t+1\right)\left|-\frac{1}{2}\left(N_{t}-1\right)\right), \end{array}$$

where equation $\sum_{i,j\in V(t)}k_{i}^{\left(t\right)}\Omega_{i,j}^{\left(t\right)}=\frac{1}{2}\sum_{i,j\in V(t)}\left(k_{i}^{\left(t\right)}+k_{j}^{\left(t\right)}\right)\Omega_{i,j}^{\left(t\right)}=\frac{1}{2}K_{V_{t},V_{t}}^{+}\left(t\right)$ is used in the above equation, and combining the three obtained term expressions leads to

(A16) $$K_{V_{t+1},V_{t+1}}^{+}\left(t\right)=\frac{5}{2}K_{V_{t},V_{t}}^{+}\left(t\right)+5K_{V_{t},V_{t}}^{*}\left(t\right)+\frac{105}{4}\times 5^{2t}-\frac{13}{2}\times 5^{t}-\frac{3}{4},$$

Equation (21) gives the exact value of $K_{V_{t},V_{t}}^{*}\left(t\right)$. Considering a initial value $K_{V_{0},V_{0}}^{+}\left(0\right)=4$, we can obtain the solution of $K_{V_{t},V_{t}}^{+}\left(t\right)$ shown in Equation (22).
